# Chemical Composition of *Impatiens textori* Miq. Flower Absolute and Its Potential Wound Repair and Anti-Melanogenesis-Promoting Activities in Skin Cells

**DOI:** 10.3390/ph15111397

**Published:** 2022-11-13

**Authors:** Yu Rim Won, Kyung Jong Won, Do Yoon Kim, Mi Jung Kim, Bok Sil Hong, Hwan Myung Lee

**Affiliations:** 1 Division of Cosmetic and Biotechnology, College of Life and Health Sciences, Hoseo University, Asan 31499, Korea; 2 Department of Physiology and Medical Science, School of Medicine, Konkuk University, Seoul 05029, Korea; 3 Korea Essential Oil Resource Research Institute, Hoseo University, Asan 31499, Korea; 4 Department of Nursing, Life Science Research Center, Cheju Halla University, Jeju 63092, Korea

**Keywords:** *Impatiens textori* Miq., absolute, skin wound healing, melanin biosynthesis, keratinocyte, melanoma cell

## Abstract

*Impatiens textori* Miq. (ITM; family Balsaminaceae) is a traditional medicinal plant with many biological activities, which include anti-allergic, anti-inflammatory, and anti-pruritic properties. However, it remains to be determined whether ITM affects biological activities in the skin. Thus, we investigated the effects of ITM flower absolute (ITMFAb) extract on the biological activities of skin, especially those related to skin wound repair and whitening. ITMFAb was extracted with hexane, and its composition was determined through GC/MS. The biological activities of ITMFAb on HaCaT keratinocytes and B16BL6 melanoma cells were analyzed using a water-soluble tetrazolium salt, 5-bromo-2′-deoxyuridine incorporation, a Boyden chamber, an ELISA, a sprouting assay, and by immunoblotting. These analyses were performed in a range of ITMFAb concentrations that did not inhibit the viability of the cells (HaCaT, ≤400 µg/mL; B16BL6, ≤200 µg/m). Forty components were identified in ITMFAb. ITMFAb stimulated proliferation, migration, sprout outgrowth, and type I and IV collagen synthesis and upregulated the activations of ERK1/2, JNK, p38 MAPK, and AKT in HaCaT cells. In addition, ITMFAb attenuated the serum-induced proliferation of B16BL6 cells. ITMFAb inhibited melanin synthesis, tyrosinase activity, and expressions of MITF and tyrosinase in α-MSH-exposed B16BL6 cells. These findings indicate that ITMFAb has beneficial effects on wound repairing and whitening-linked responses in the skin and suggest the potential use of ITMFAb as a natural material for the development of skin wound repair and whitening agents.

## 1. Introduction

Skin wounds need to be repaired rapidly and effectively to facilitate skin regeneration because the skin acts as a barrier to protect the body from harmful environmental factors. The skin healing process involves complex physiological responses that consist of three overlapping phases, viz. inflammation, proliferation, and remodeling [1], which involve interactions between various types of cells (e.g., keratinocytes, immune cells, and fibroblasts) and response mediators, such as growth factors, chemokines, and extracellular matrix (ECM)) in dermal and epidermal layers [2,3]. When skin is injured, keratinocytes (the predominant cell type in the epidermis) proliferate at wound margins and migrate to the wound area to support re-epithelialization [4]. These cells are the main contributors to re-epithelialization during the proliferation phase of wound healing [4], and their activities are regulated by various actors such as growth factors (transforming and epidermal growth factors), integrin, ECM (collagens), and metalloproteases [5]. Abnormal or disrupted wound healing may delay healing and impair normal skin functions, and thus, therapeutic agents are needed to promote the complete healing of skin wounds.

Melanin, a determinant of skin color, plays an important role in pigmenting and protecting skin from damage [6]. However, excessive production of melanin in the skin can cause hyperpigmentation-related disorders such as freckles, solar lentigo, and melanoma [7]. Melanin is synthesized in melanosomes in melanocytes via complex enzymatic and chemical reactions induced by various stimuli, such as UV light, toxic agents, or α-melanocyte-stimulating hormone (MSH) [8]. Furthermore, melanin synthesis in melanocytes is closely associated with activations of melanogenesis-associated regulatory proteins such as tyrosinase, the key tyrosinase-related protein-1 (TRP-1), and TRP-2 [7]. In particular, tyrosinase, the rate-limiting enzyme in melanogenesis, catalyzes the hydroxylation of L-tyrosine to 3,4-dihydroxy-L-phenylalanine (L-DOPA) and the transformation of L-DOPA to L-DOPA quinone, which is subsequently converted into DOPA chrome [9]. TRP-2 plays a crucial role in the conversion of DOPA chrome to 5,6-dihydroxyindole-2-carboxylic acid (DHICA), and TRP-1 promotes the oxidization of DHICA to produce indole-2-carboxylic acid (IQCA) [9]. These enzymatic processes are also regulated by microphthalmia-associated transcription factor (MITF), which is a key regulator of melanogenesis [7]. Many studies have focused on the development of anti-melanogenic agents and skin-whitening agents [8]. However, the agents developed so far have adverse effects such as dermatitis and erythema [8]. Accordingly, recent research interest has shifted to the identification of safer, more effective natural materials with anti-melanogenic and skin-whitening effects.

*Impatiens textori* Miq. (ITM; family Balsaminaceae) is distributed in Korea, Northeast China, and Japan [10,11] and is a traditional medicinal plant that was used as a detoxicant, and to treat carbuncles, contusions, superficial infections, and fingernail inflammations [12,13,14]. Leaf extracts of ITM have antioxidant and antiproliferative effects on cancer cells [15], and the whole plant and flower extracts of ITM possess anti-allergic, anti-pruritic, and anti-inflammatory effects in mouse models and inflammatory cells [11,13,14]. However, no previous study has investigated the effects of ITM or its extracts on skin wound healing or whitening. In this study, we aimed to explore the wound-healing properties of ITM flower absolute extract (ITMFAb) in human keratinocytes (HaCaT cells) and to investigate the effect of ITMFAb on skin whitening-related responses in melanoma cells (B16BL6 cells). In addition, we suggest a mechanistic basis for these effects

## 2. Results

### 2.1. Chemical Composition of ITMFAb

**Figure 1:**
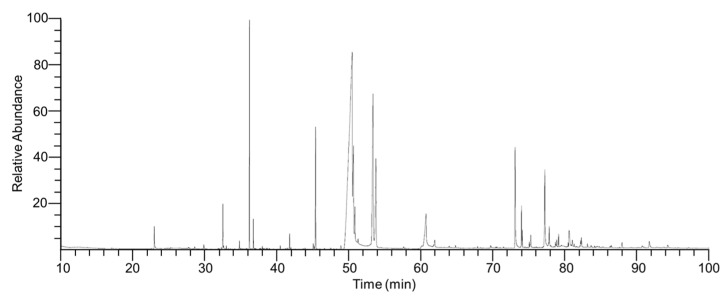
GC/MS total ion chromatogram of *Impatiens textori* Miq. flower absolute. Peaks represent the 40 identified compounds listed in Table 1.

Gas chromatography/mass spectrometry (GC/MS) revealed 40 compounds in ITMFAb (Figure 1 and Table 1). Palmitoleic acid (55.41%, expressed as a percentage of total peak area) had the highest content, and the contents of the eight next most abundant compounds were as follows: palmitelaidic acid, TMS (15.85%), methyl undecanoate (4.34%), 1-tetracosanol (4.20%), linolenic acid (4.02%), docosanol (3.83%), tetradecanoic acid, trimethylsilyl ester (3.57%), 22-tricosenoic acid (1.40%), and geraniol (0.92%) (Table 1).

### 2.2. Effects of ITMFAb on Keratinocyte Proliferation and Migration

**Figure 2:**
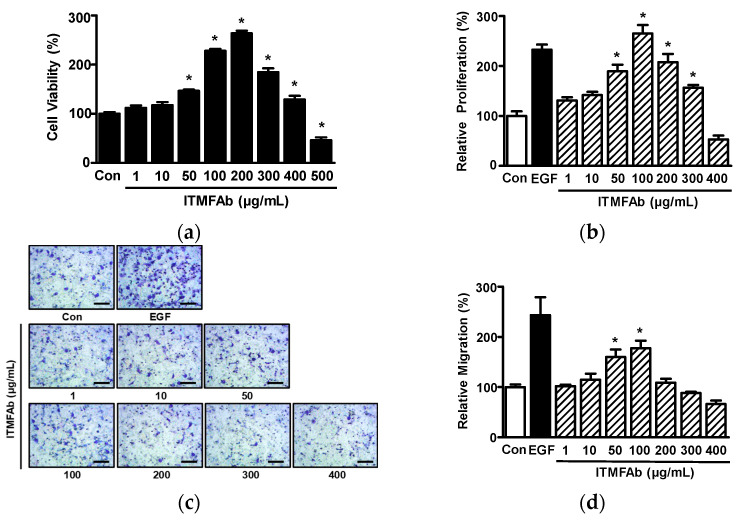
Effects of *Impatiens textori* Miq. flower absolute on the viability of HaCaT cells, proliferation, and migration. (**a**) Cell viability. HaCaT cells were incubated with *Impatiens textori* Miq. flower absolute (ITMFAb; 1–500 μg/mL) for 48 h and cell viabilities were evaluated using the WST assay (*n* = 5) (**b**) Cell proliferation. HaCaT cells were treated with ITMFAb (1–400 µg/mL) for 48 h, and a BrdU incorporation assay was performed to measure proliferation. Recombinant human epidermal growth factor (EGF: 50 ng/mL): positive control. Cell proliferation levels are presented as percentages of those of non-treated controls (Con). Data are expressed as means ± standard errors of means (SEMs) of non-treated controls (*n* = 5). * *p* < 0.05 vs. non-treated cells. (**c**) Representative image of cell migration results. HaCaT cells were treated with ITMFAb (1–400 µg/mL) for 210 min, and cell migration was assessed using a Boyden chamber. Migrated cells are shown as violet spots. Scale bar = 50 μm. (**d**) Graph obtained from panel (**c**). Recombinant human epidermal growth factor (EGF: 1 ng/mL): positive control. Cell migratory levels are presented as percentages of non-treated controls (Con). Data are expressed as means ± SEMs of non-treated controls (*n* = 4). * *p* < 0.05 vs. non-treated controls.

Keratinocyte migration and proliferation are known to influence the healing process [16], and thus to determine the involvement of ITMFAb in skin wound healing, we examined the effects of ITMFAb on the proliferation and migration of keratinocytes. Initially, we assessed the effects of different ITMFAb concentrations (1–500 µg/mL) on the viability of HaCaT cells using a water-soluble tetrazolium salt (WST) assay. ITMFAb (50–400 μg/mL) significantly increased viability as compared with non-treated controls, and this peaked at 200 μg/mL (263.52 ± 5.58% of non-treated controls). However, at 500 μg/mL ITMFAb significantly reduced cell viability versus controls (Figure 2a), and thus, subsequent experiments were conducted using concentrations of ≤400 µg/mL. Cell proliferation after treating HaCaT cells with ITMFAb (1–400 μg/mL) was analyzed using a 5-bromo-2′-deoxyuridine (BrdU) incorporation assay. ITMFAb treatment significantly increased HaCaT cell proliferation at concentrations of 50 to 300 μg/mL and showed the greatest effect at a concentration of 100 μg/mL (265.19 ± 16.93% of non-treated controls) (Figure 2b).

The effect of ITMFAb (1–400 μg/mL) on the migration of HaCaT cells was also evaluated using a Boyden chamber assay. Treatment of HaCaT cells with ITMFAb significantly increased cell proliferation at 50 and 100 μg/mL (159.85 ± 14.97% and 177.75 ± 14.95%, respectively, versus non-treated controls) (Figure 2c,d).

### 2.3. Effect of ITMFAb on Keratinocyte Sprout Outgrowth

**Figure 3:**
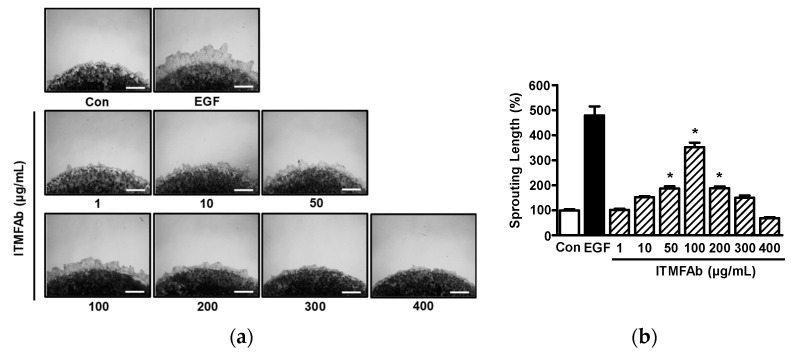
Effect of *Impatiens textori* Miq. flower absolute on sprout outgrowth by HaCaT cells. Collagen-mixed HaCaT cells were spotted on a 24-well plate. Spots were incubated in the presence or absence of *Impatiens textori* Miq. flower absolute (ITMFAb; 1–400 μg/mL) for 48 h and then stained with Diff-Quick solution. Images were taken using a microscope. Recombinant human epidermal growth factor (EGF: 50 ng/mL): positive control. (**a**) Representative result images. Scale bar = 50 μm. (**b**) Graph of the results obtained. Sprout formation levels are expressed as percentages of those in non-treated controls (Con). Data are expressed as the means ± SEMs of non-treated controls (*n* = 9). * *p* < 0.05 vs. non-treated controls.

The effects of ITMFAb on keratinocyte proliferation and migration described above were confirmed using the collagen sprout assay, which is commonly used for this purpose [16]. Treatment with ITMFAb (1–400 μg/mL) significantly increased HaCaT cell sprouting at concentrations of 50 to 200 μg/mL, and this peaked at 100 μg/mL (352.21 ± 18.01% of non-treated controls) (Figure 3).

### 2.4. ITMFAb-Induced Changes in the Activations of Kinases in HaCaT Cells

**Figure 4:**
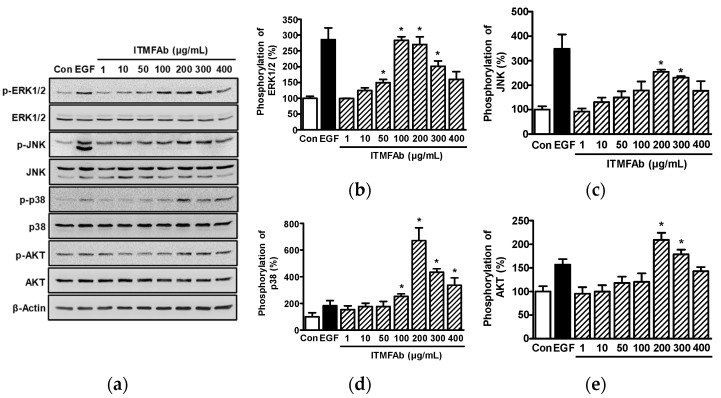
Effect of *Impatiens textori* Miq. flower absolute on the activation of kinases in HaCaT cells. HaCaT cells were incubated with or without *Impatiens textori* Miq. flower absolute (ITMFAb; 1–400 μg/mL) for 10 min and cell lysates were immunoblotted with the indicated antibodies. (**a**) Representative images of immunoblotting results (**b**–**e**) Graphs of phosphorylated ERK1/2 (**b**), JNK (**c**), p38 MAPK (**d**), and AKT expression levels (**e**) obtained from panel (**a**). Kinase phosphorylation levels are expressed as percentages of those in non-treated controls (Con). Recombinant human epidermal growth factor (EGF: 5 ng/mL): positive control. Data are presented as means ± SEMs (*n* = 3/protein). * *p* < 0.05 vs. non-treated controls. p-ERK1/2, phosphorylated ERK 1/2; p-JNK, phosphorylated JNK; p-p38, phosphorylated p38 MAPK; p-AKT, phosphorylated AKT.

Kinases such as mitogen-activated protein kinases (MAPKs) and serine/threonine-specific protein kinases (AKT) act as signaling molecules for the migration and proliferation of HaCaT cells [17,18]. Thus, we investigated whether these signaling molecules are involved in the ITMFAb-induced migration and proliferation of HaCaT cells by Western blotting. ITMFAb significantly enhanced the activations of extracellular signal-regulated kinase1/2 (ERK1/2) at 50 to 300 μg/mL (Figure 4a,b), c-Jun N-terminal kinase (JNK) (Figure 4a,c) and AKT (Figure 4a,e) at 200 and 300 μg/mL, and p38 MAPK (Figure 4a,d) at 100 to 400 μg/mL. ITMFAb-induced ERK1/2 activation peaked at an ITMFAb concentration of 100 μg/mL (283.09 ± 11.78% of non-treated controls, Figure 4b), and the activation levels of JNK, p38 MAPK, and AKT peaked at ITMFAb concentrations of 200 μg/mL (254.31 ± 8.78% (Figure 4c), 670.83 ± 96.02% (Figure 4d)), and 209.29 ± 15.14% (Figure 4e), respectively, of the non-treated controls).

### 2.5. ITMFAb-Induced Changes in Collagen Synthesis in HaCaT Cells

**Figure 5:**
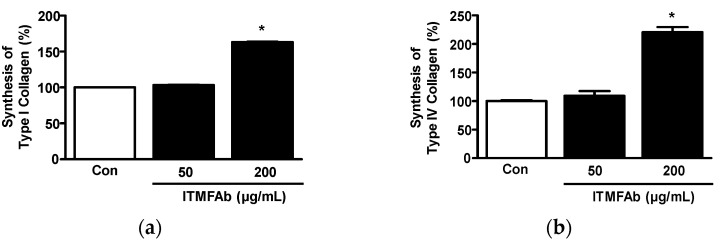
Effects of *Impatiens textori* Miq. flower absolute on the syntheses of type I and Ⅳ collagens. HaCaT cells were cultured with or without *Impatiens textori* Miq. flower absolute (ITMFAb; 50 and 200 μg/mL) for 48 h and sandwich ELISA was performed in conditioned media using anti-type I (*n* = 3; (**a**)) or anti-type IV collagen antibody (*n* = 3; (**b**)). Collagen levels in conditioned media in non-treated controls (Con) were considered 100%. Data are expressed as means ± SEMs. * *p* < 0.05 vs. non-treated controls.

Collagen synthesis plays an important role in all skin healing processes, and types I and IV collagen participate in skin recovery and skin membrane formation [17,19,20]. Thus, to determine whether ITMFAb affects collagen synthesis by keratinocytes, we performed sandwich ELISA on a conditioned medium that was collected by incubating HaCaT cells in the presence or absence of ITMFAb. Treatment with ITMFAb (50 and 200 μg/mL) significantly elevated both type I and IV collagen synthesis at 200 μg/mL (163.24 ± 0.59% (Figure 5a) and 220.47 ± 9.10% (Figure 5b), respectively, versus non-treated controls).

### 2.6. Effects of ITMFAb on B16BL6 Melanoma Cells

**Figure 6:**
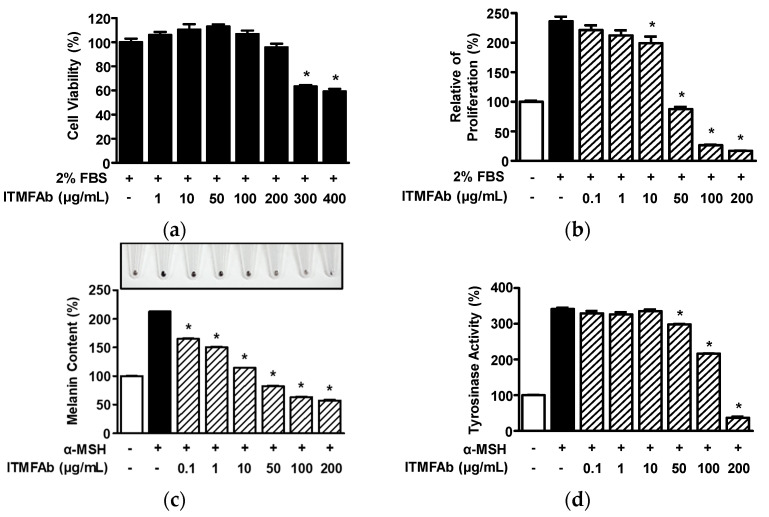
Effects of *Impatiens textori* Miq. flower absolute on skin whitening-linked responses in B16BL6 melanoma cells. (**a**) Cell viability. B16BL6 melanoma cells in DMEM containing 2% FBS were treated with or without *Impatiens textori* Miq. flower absolute (ITMFAb; 1–400 μg/mL) at 37 °C for 48 h. Cell viability levels were determined using a WST assay (*n* = 5). Levels in the 2% FBS alone-treated state were considered 100%. * *p* < 0.05 vs. cells treated with 2% FBS. (**b**) Cell proliferation. B16BL6 melanoma cells were cultured in DMEM with or without 2% FBS in the presence or absence of ITMFAb (0.1–200 μg/mL) at 37 °C for 48 h. Proliferations were measured using the BrdU incorporation assay described in Materials and Methods (*n* = 5). Data are expressed as percentages of responses in the quiescent state. * *p* < 0.05 vs. cells treated with 2% FBS. (**c**,**d**) Melanin contents and tyrosinase activities. B16BL6 melanoma cells were incubated in Dulbecco’s modified eagle medium (DMEM) (containing 2% FBS) with or without α-MSH (200 nM) in the presence or absence of ITMFAb (0.1–200 μg/mL) at 37 °C for 48 h. Melanin contents ((**c**); *n* = 3) and tyrosinase activities ((**d**); *n* = 3) were measured as described in Materials and Methods. The upper image in panel C shows a representative result image. Results are expressed as mean percentages ± SEMs of levels in non-treated controls (2% FBS alone). * *p* < 0.05 vs. cell treated with α-MSH.

Initially, we evaluated the effect of ITMfAb on B16BL6 cell viability in the presence of 2% fetal bovine serum (FBS) using the WST assay. ITMFAb (1–400 μg/mL) had no significant effect at concentrations of 1 to 200 μg/mL but decreased viability at 300 and 400 μg/mL (Figure 6a). Thus, subsequent experiments on B16BL6 cells were performed at ITMFAb concentrations of ≤200 μg/mL. When we examined the effect of ITMFAb on B16BL6 melanoma cell proliferation, FBS (2 %) increased B16BL6 proliferation (236.46 ± 7.55 % versus non-treated controls; Figure 6b). However, this increase was inhibited by ITMFAb at 10 to 200 μg/m and peaked at an ITMFAb concentration of 200 μg/mL (17.00 ± 0.38 % of non-treated controls; Figure 6b). Finally, we evaluated the effects of ITMFAb on melanin production and tyrosinase activity in B16BL6 melanoma cells exposed to α-MSH (an inducer of melanin synthesis). α-MSH (200 nM) increased melanin production (213.12 ± 0.60% versus non-treated controls), and ITMFAb at 0.1–200 μg/mL significantly and concentration-dependently inhibited this α-MSH-induced increase. The inhibitory effect of ITMFAb peaked at 200 μg/mL (57.48 ± 0.86% of non-treated controls) (Figure 6c). On the other hand, α-MSH (200 nM) increased tyrosinase activity (341.45 ± 4.12% versus non-treated controls), and ITMFAb (50–200 μg/mL) significantly reduced this α-MSH-induced increase and had a maximum effect at a 200 μg/mL (36.90 ± 3.60 % of non-treated controls) (Figure 6d).

### 2.7. Effects of ITMFAb on Melanogenesis-Regulatory Molecules in B16BL6 Melanoma Cells

**Figure 7:**
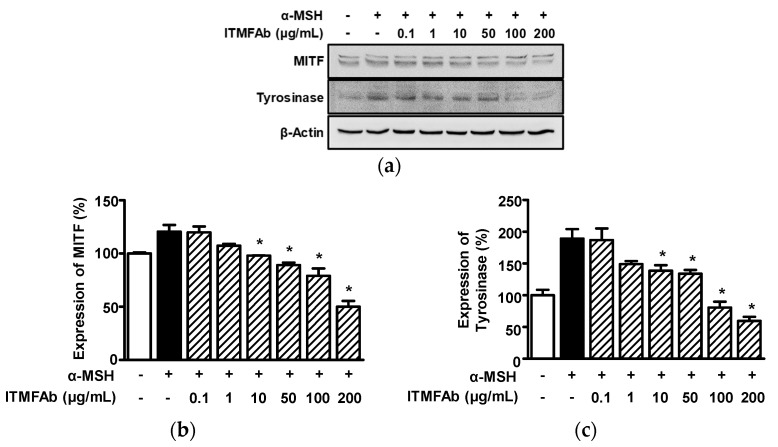
Effects of *Impatiens textori* Miq. flower absolute on melanogenesis-associated proteins in B16BL6 melanoma cells. (**a**) Representative images. B16BL6 melanoma cells were pretreated with DMEM (containing 2% FBS) in the presence or absence of α-MSH (200 nM) for 10 min or incubated with or without α-MSH (200 nM) in the presence or absence *Impatiens textori* Miq. flower absolute (ITMFAb; 0.1–200 μg/mL) for 5 min. Cell lysates were immunoblotted with the antibodies described in Materials and Methods. (**b**,**c**) Graphs MITF (*n* = 3; (**b**)) and tyrosinase (*n* = 3; (**c**)) expression levels. Expression levels of each protein are expressed as percentages of levels in non-treated controls (2% FBS alone). Data are presented as means ± SEMs. * *p* < 0.05 vs. cell treated with α-MSH alone.

We also evaluate the effects of ITMFAb on melanin biosynthesis-associated proteins in B16BL6 cells in the presence of α-MSH by Western blotting. First, we tested the effects of ITMFAb on MITF and tyrosinase expressions in B16BL6 cells in the presence of α-MSH. α-MSH (200 nM) increased the expressions of MITF (120.52 ± 6.29% versus non-treated controls, Figure 7a,b) and tyrosinase increased it (189.37 ± 14.89% versus non-treated controls, Figure 7a,c). ITMFAb (0.1 to 200 μg/mL) attenuated the α-MSH-induced expressions of MITF and tyrosinase in a concentration-dependent manner, and these effects were significant at ITMFAb concentrations of 10 to 200 μg/mL and peaked at 200 μg/mL (50.07 ± 5.31% (Figure 7a,b) and 59.54 ± 6.60% (Figure 7a,c), respectively, of non-treated control levels).

## 3. Discussion

Skin wound healing is a lengthy process and is prone to risk factors such as infection. Accordingly, many studies have been conducted with the aim of identifying materials that promote wound healing and skin regeneration, and several plants and plant extracts have been shown to promote wound healing with fewer adverse effects [21,22]. Thus, many researchers have investigated the wound-healing effects of plants and plant extracts [23,24]. In the present study, we investigated the effects of ITMFAb on wound healing-related responses in keratinocytes. ITMFAb promoted the proliferation and migration of HaCaT keratinocytes and induced sprout outgrowth from HaCaT cells in a collagen sprouting assay, which supported its observed cell migratory and proliferative effects [25]. Keratinocyte proliferation and migration are activated by skin damage, and these activations result in re-epithelialization, which is essential for wound healing [5]. Therefore, our proliferation and migration findings indicate that ITMFAb might contribute to skin wound healing responses. In the present study, GC/MS analysis revealed the presence of 40 compounds in ITMFAb. Of these, α-bisabolol, lupeol, botulin, oleic acid, and tocopherols have been reported to promote wound healing-linked responses in mice and rats by inhibiting inflammatory responses and stimulating angiogenesis and the expressions of growth factors [26,27,28,29,30,31]. Reports indicate lupeol and betulin-based oleogel improve wound healing by stimulating keratinocyte migration [30,32], which implies that these compounds may have contributed to ITMFAb-induced HaCaT migration. On the other hand, other compounds found in ITMFAb have not been reported to induce skin wound healing responses. Therefore, further studies are needed to clarify the effects of these other compounds on wound-healing responses in keratinocytes, which may contribute to skin wound healing.

MAPKs are important intracellular signaling molecules that modulate cellular proliferation and migration during wound healing [33]. ERK1/2, JNK, and p38 MAPK are the most widely MAPKs and participate in migration and proliferation-mediating signaling pathways in keratinocytes [33,34]. In keratinocytes, migration and proliferative activities were enhanced by increasing ERK1/2 activation [34] and attenuated by reducing activation [35]. Furthermore, keratinocyte proliferation and migration were upregulated by increasing p38 MAPK levels [36]. On the other hand, JNK has been reported to have variable effects on the regulations of keratinocyte migration and proliferation; for example, keratinocyte migration and proliferation have been reported to be increased [34] and to be unaffected by JNK activation [37]. In the present study, ITMFAb induced the phosphorylations of ERK1/2, p38 MAPK, and JNK in HaCaT cells. We previously showed that these MAPKs might be associated with keratinocyte migration and proliferation [38], which implies MAPKs play positive roles in ITMFAb-induced HaCaT cell migration and proliferation. Additionally, AKT is an important mediator of keratinocyte migration and proliferation [39,40,41], and increased AKT activation has been reported to facilitate HaCaT cell migration and proliferation [34]. Interestingly, we observed that ITMFAb enhanced AKT activation in HaCaT cells. These observations suggest that ITMFAb induces migratory and proliferative responses in keratinocytes by activating the AKT and MAPK signaling pathways.

Collagen functions as a scaffold in skin tissues, and it is also associated with cell adhesion, migration, and proliferation [42]. Furthermore, collagen is essential for all skin healing stages; for example, in the proliferative phase, collagen contributes to the motility of keratinocytes [5,43]. For these reasons, collagen is used as an adjuvant therapy to promote skin healing and enhance keratinocyte proliferation [44]. Collagen type I (interstitial collagen) and type IV (basement membrane collagen) have been reported to enhance the migratory and proliferative activities of keratinocytes [45] and were produced and secreted by HaCaT cells stimulated with plant extracts [17,25]. In addition, these collagen types play roles in skin recovery [46] and skin membrane formation [43,47], respectively. In the present study, we found that collagen type I and IV levels were upregulated in the conditioned medium of HaCaT cells exposed to ITMFAb, indicating that ITMFAb induces the syntheses of these collagens in keratinocytes.

Melanin is synthesized in melanocytes to protect the skin from external stressors, especially UV light, but on the other hand, excessive melanin production is problematic [8]. Thus, many attempts have been made to inhibit melanin biosynthesis. In this context, we evaluated the inhibitory effects of ITMFAb on melanin biosynthesis-related events in B16BL6 melanoma cells. Melanocyte survival, proliferation, and differentiation positively affect melanin biosynthesis [48], and thus the inhibition of melanocyte proliferation suppresses melanogenesis [49]. Notably, we found ITMFAb inhibited B16BL6 melanoma cell proliferation induced by 2% FBS, implying that ITMFAb may have an inhibitory effect on melanogenesis through the inhibition of melanocyte proliferation. MITF acts as a major modulator of tyrosinase [7,50]. In B16 melanoma cells, melanin production and the protein levels of tyrosinase were suppressed by reducing MITF expression [51]. In the present study, ITMFAb decreased MITF expression and tyrosinase expression in B16BL6 melanoma cells exposed to α-MSH. This result implies that ITMFAb may influence melanin production via MITF-mediated tyrosine action. Tyrosinase acts as an initial and rate-limiting enzyme that participates in the first step of melanin biosynthesis [9], and tyrosinase activity and level are directly correlated with melanogenesis [52]. In α-MSH-stimulated B16BL6 melanoma cells, inhibitions of tyrosinase expression and activity reduced melanin production [53]. Similarly, in the present study, ITMFAb attenuated tyrosinase expression and activity and melanin production in α-MSH-stimulated B16BL6 melanoma cells. Therefore, our observations suggest that ITMFAb may negatively regulate melanin production by suppressing MITF-mediated tyrosinase action or by inhibiting tyrosinase action. As mentioned above, we identified 40 compounds in ITMFAb, and of these, several have been reported to induce skin whitening by inhibiting melanogenesis-related responses in melanocytes. In particular, geranic acid, α-bisabolol, palmitoleic acid, linolenic acid, ethyl linolenate, and oleic acid were reported to decrease melanin production, tyrosinase activity, tyrosinase expression, or MITF expression [54,55,56,57,58,59,60]. Therefore, these compounds might contribute to the suppression of melanogenesis-related responses by ITMFAb in melanocytes. However, whether these or other compounds are related to ITMFAb inhibition of melanogenesis in melanocytes needs to be clarified.

## 4. Materials and Methods

### 4.1. Materials

Penicillin/streptomycin (P/S), trypsin–ethylenediamine tetra-acetic acid (EDTA), and FBS were purchased from Gibco BRL (Gaithersburg, MD, USA) and Dulbecco’s modified eagle medium (DMEM) and phosphate-buffered saline (PBS) from Welgene (Daegu, Korea). Triton X-100, L-DOPA, bovine serum albumin (BSA), α-MSH, phenylmethylsulfonyl fluoride, NaOH, 10× DMEM, Tween-20 and dimethyl sulfoxide (DMSO) were from MilliporeSigma (St. Louis, MO, USA). Recombinant human epidermal growth factor (EGF: purity > 97%) was obtained from R&D Systems (Minneapolis, MN, USA). EZ-CyTox kits were supplied by DoGenBio (Seoul, Korea) and type I collagen by BD Bioscience (Franklin Lakes, NJ, USA). The antibodies used were as follows: anti-ERK1/2, anti-phospho ERK1/2, anti-AKT, anti-phospho AKT, anti-JNK, anti-phospho JNK, anti-p38 MAPK, anti-phospho p38 MAPK, anti-rabbit IgG, anti-MITF, and anti-mouse IgG (all from Cell Signaling, Beverly, MA, USA); polyclonal anti-type I, and IV collagen, monoclonal anti-type I and IV collagen (Abcam; Cambridge, UK); β-actin (MilliporeSigma); and anti-tyrosinase (Santa Cruz Biotechnology, CA, USA).

### 4.2. Preparation of Impatiens textori Miq. Flower Absolute

ITM flowers growing in Hanaro Farm (Songji-myeon, Jeollanam-do, Korea; 34°23′00.4′′ N 126°33′59.0′′ E) were collected on 12 September 2018 and identified by Jong-Cheol Yang (Baekdudaegan National Arboretum, Bonghwa-gun, Korea). A voucher specimen (No. IT-0002) was kept at the Herbarium of the College of Life and Health Science (Hoseo University, Korea). Absolute was extracted by solvent extraction method as in a previous report [16]. In brief, ITM flowers (4.05 kg) were immersed in 15 L of hexane (Samchun, Pyeongtaek, Korea) at room temperature (RT) for 1 h. Extracts were collected, and the hexane removal was carried out by a rotary evaporator (Eyla, Tokyo, Japan) at 25 °C under vacuum to obtain a dark yellow waxy residue (concrete). This residue was then dissolved in ethanol (99.5%; Samchun Chemicals, Pyungtaek, Korea), left at −20 °C for 12 h, filtered through a sintered funnel, and then evaporated at 35 °C to remove ethanol, leaving a light-yellow anhydrous wax (ITMFAb; 3.38 g, yield 0.083%, *w*/*w*). The ITMFAb obtained was stored at −80 °C until required.

### 4.3. Analysis and Identification of Compounds in ITMFAb

ITMFAb was analyzed by the National Instrumentation Center for Environmental Management (NICEM, Seoul National University, Korea), and its components were identified by GC/MS. GC/MS data were acquired using a TRACE 1310 GC attached to an ISQ LT single quadrupole mass spectrometer (Thermo Scientific, Waltham, MA, USA). Derivatized samples were separated on a DB-5MS column (60 m × 0.25 mm, 0.25 μm; Agilent Technologies, USA) at a constant flow rate of 1 mL/min using the following program; 50 °C for 5 min, 50 to 65 °C at 10 °C/min, 65 to 210 °C at 5 °C/min, 210 to 310 °C at 20 °C/min, and 310 °C for 10 min. Masses from 35–550 *m*/*z* were scanned, and the data acquisition rate was 0.2 scans/s. Transfer line and ion source temperatures were 300 and 270 °C, respectively. Compounds were identified by comparing spectra and retention indices (RI) with reference standards in the NIST/NIH/EPA mass spectral library (NIST 11, version 2.0 g) and by comparing retention times and spectra with those of commercially available standards. A standard solution of n-alkanes (C_7_–C_30_) was used to determine RIs.

### 4.4. Cell Culture

The human keratinocyte cell line (HaCaT cells) was obtained from the National Institute of Korean Medicine Development (NIKOM, Gyeongsan, Korea), and the murine melanoma cell line (B16BL6 cells) from the Korean Cell Line Bank (KCLB, Seoul, Korea). Both cell types were maintained in DMEM containing 10% FBS and 1% P/S at 37 °C in a humidified 95% air/5% CO_2_ atmosphere. Cells were cultured until 70–80% confluent for experiments.

### 4.5. Cell Viability Assays

Cell viability was analyzed using a WST assay using the EZ-CyTox kit (DoGenBio). Cells (HaCaT cells 3 × 10^3^ cells/well; B16BL6 cells 2 × 10^3^ cells/well) were seeded into 96-well microtiter plates and then treated with different concentrations of ITMFAb (dissolved in DMEM containing 0.5% DMSO for HaCaT cells and in DMEM containing 0.5% DMSO and 2% FBS for B16BL6 cells) for 48 h. The cells were then incubated with EZ-CyTox reagent (10 μL/well) for 30 min at 37 °C. Absorbances were measured at 450 nm using an enzyme-linked immunosorbent assay (ELISA) reader (Synergy 2, Bio-Tek Instruments, Winooski, VT, USA).

### 4.6. Proliferation Assays

Cell proliferation was analyzed using a BrdU (5-bromo-2′-deoxyuridine) incorporation assay using a cell proliferation enzyme-linked immunosorbent assay (ELISA) (BrdU kit; Roche, Indianapolis, Indiana, USA). Briefly, Cells (HaCaT (3 × 10^3^ cells/well) or B16BL6 cells (2 × 103 cells/well)) were plated in 96-well plates, incubated for 12 h, treated with various concentrations of ITMFAb (dissolved in DMEM containing 0.5% DMSO) or EGF (50 ng/mL) for 36 h, and labeled with BrdU-labeling solution (10 μM) for an additional 12 h at 37 °C. After removing the culture medium, cells were fixed and incubated to denature DNA using FixDenat solution from the BrdU kit for 30 min at RT and then incubated with peroxidase-labeled anti-BrdU monoclonal antibody incubated for 90 min at RT. The luminescence of the reaction product was measured using a luminometer (Synergy 2).

### 4.7. Migration Assay

Cell migration assay was performed using a 48-well Boyden microchemotaxis chamber (Neuro Probe Inc., Gaithersburg, MD, USA), as in a previous report [16]. Briefly, lower chamber wells were filled with DMEM containing 0.1% BSA and EGF (1 ng/mL) or different concentrations of ITMFAb. A membrane (Neuro Probe, Cabin John, MD, USA) coated with type Ι collagen (0.1 mg/mL) was then laid over lower chamber wells, and upper chamber wells were loaded with HaCaT cells (5 × 10^4^ cells/well) in DMEM containing 0.1% BSA. Chambers were assembled and incubated for 210 min at 37 °C. Membranes were then removed, fixed, and stained using Diff-Quick (Baxter Healthcare, Miami, FL, USA). The cells migrating to lower membrane surfaces were counted using an optical microscope (Carl Zeiss, Jena, Jena, Thuringen, Germany) (×200).

### 4.8. Collagen Sprout Assay

Collagen sprout assays were performed to simultaneously analyze both cell migration and proliferation. HaCaT cells (2.5 × 10^7^ cells/mL) were mixed with type I collagen, 10× DMEM medium, and 1 N NaOH (pH 7.2) and placed in were in spot form on the wells of 24-well cell culture plate. After drying for 20 min, the spots were treated with or without ITMFAb or EGF (50 ng/mL) and incubated at 37 °C in a CO_2_ incubator for 48 h. Spots were fixed and stained using a Diff–Quik solution, and images were then taken using an optical microscope (Carl Zeiss) (×100). Lengths of sprouts were measured using Image J software (NIH, Bethesda, MD, USA).

### 4.9. Collagen Synthesis Assay

A collagen synthesis assay was performed using ELISA, as in a previous report [16]. Briefly, HaCaT cells (5 × 10^5^ cells/well)were seeded in 100 mm cell culture dishes and treated with various concentrations of ITMFAb for 48 h at 37 °C. Cultured media were centrifuged sequentially at 500, 800, and 1000× *g* for 10 min. The supernatants (conditioned media; 100 μL/well) obtained were loaded into 96-well microtiter plates coated with type I or IV collagen monoclonal antibody (capture antibody), and incubated with biotin-conjugated collagen type I or IV polyclonal antibody (dilution 1:2000 in 1% BSA/PBS) for 90 min at RT. Each well was washed with PBS, loaded with streptavidin-horseradish peroxidase conjugate (Roche) (diluted 1:5000 in 1% BSA/PBS) for 1 h at RT, and washed again with PBS. Enhanced chemiluminescence solution (Thermo Fisher Scientific, Waltham, MA, USA) was then added, and the luminescence of the reaction product was measured using a luminometer (Synergy 2).

### 4.10. Western Blotting

Cells were lysed using RIPA (radioimmunoprecipitation assay) buffer (Cell Signaling), followed by centrifugation at 17,000× g for 15 min at 4 °C. Protein concentrations in supernatants were measured using DC protein assay reagents (Bio-Rad Laboratories, Hercules, CA, USA). In addition, proteins (60–120 μg/lane) were separated by SDS-PAGE on 10% acrylamide gel and transferred to polyvinylidene fluoride membranes (MilliporeSigma) at 4 °C. After blocked with 3% skim milk solutions at RT for 2 h, membranes were washed with PBS containing 0.05% Tween-20, incubated with the relevant primary antibodies (1:1000–5000 dilution), and then with conjugated horseradish peroxidase at RT for 1 h. The immunoreactive protein bands were visualized using a chemiluminescence substrate and detected using a chemiluminescence imaging system (LuminoGraph, ATTO, Tokyo, Japan).

### 4.11. Melanin Content Assay

Melanin content assay was performed as in a previous report [61]. Briefly, B16BL6 cells (1 × 10^5^ cells per dish) were seeded in 60 mm dishes, cultured for 12 h, and incubated in DMEM (containing 2% FBS) with or without various concentrations of ITMFAb in the presence or absence of 200 nM α-MSH for 48 h at 37 °C. After washing with PBS, cells were lysed with lysis buffer (0.1 M sodium phosphate buffer (pH 6.8) containing 1% Triton X-100 and 0.2 mM phenylmethylsulfonyl fluoride), followed by centrifugation at 10,000× *g* for 15 min. Cell pellets collected were dissolved in 150 μL of 1 N NaOH containing 10% DMSO, incubated at 80 °C for 1 h, and pipetted to solubilize the melanin. Absorbances were measured at 405 nm using an ELISA reader (Synergy 2).

### 4.12. Tyrosinase Activity Assays

Cells were lysed using radioimmunoprecipitation assay buffer (RIPA buffer; Cell Tyrosinase activities were analyzed using the dopachrome method using L-DOPA as substrate as in a previous report [17]. Briefly, B16BL6 cells (5 × 10^5^ cells/well) were cultured in 60 mm dishes, lysed, and centrifuged, as described for the melanin content assay above. Supernatants (60 μL) and 2 mg/mL L-DOPA (140 μL) were then added to each well of a 96-well plate and incubated at 37 °C for 60 min. Absorbances of the reaction product were measured at 490 nm using an ELISA reader (Synergy 2).

### 4.13. Statistical Analysis

Results are expressed as means ± standard errors of means (SEMs). Student’s *t*-test was used to determine the statistical significance of differences between pairs of groups, and one-way analysis of variance (ANOVA) followed by Tukey’s post hoc test was used to determine the statistical significance of differences between multiple groups. The analyses were performed using GraphPad Prism (version 5.0; GraphPad Software, Inc., San Diego, CA, USA). *p* values of <0.05 were considered to indicate significant differences.

## 5. Conclusions

In the present study, we report that 40 compounds were identified in ITMFAb and that ITMFAb induced HaCaT cell proliferation, migration, and sprout outgrowth, enhanced the phosphorylations of ERK1/2, JNK, p38 MAPK, and AKT, and promoted type I and IV collagen syntheses in HaCaT cells. Furthermore, treatment of B16BL6 cells with ITMFAb reduced serum-induced proliferation and attenuated α-MSH-induced melanin production and tyrosinase activity. In addition, ITMFAb decreased α-MSH-induced MITF expression and tyrosinase expression. These findings suggest that ITMFAb might promote skin wound healing-associated responses in keratinocytes and induce skin whitening-related responses in melanocytes. Therefore, ITMFAb appears to offer a promising basis for the development of natural skin wound healing and whitening agents. However, further studies are needed to identify the bioactive components in ITMFAb primarily responsible for its skin wound healing and whitening-related effects.

## Figures and Tables

**Table 1:** Components of *Impatiens textori* Miq. flower absolute.

No	Component Name	RT ^1^	RI ^2^	Area (%)	CAS No.
1	Ethyl 3-ethoxypropionate	22.93	1225	0.66	763-69-9
2	Linalool	27.75	1256	0.08	78-70-6
3	2-Phenylethanol	28.53	1261	0.07	60-12-8
4	3,6-Nonadien-1-ol, (E,Z)-	29.82	1269	0.16	56805-23-3
5	Geraniol	32.46	1286	0.92	106-24-1
6	[(3,7-Dimethylocta-2,6-dien-1-yl)oxy] (trimethyl) silane	34.77	1301	0.15	72237-31-1
7	Methyl undecanoate	36.16	1312	4.34	1731-86-8
8	Geranic acid TMS	36.7	1317	0.53	97779-61-8
9	2-tert-Butyl-4-isopropyl-5-methylphenol	37.93	1327	0.10	None
10	Undecanoic acid, TMS derivative	38.55	1333	0.03	146846-83-5
11	Dodecanoic acid, trimethylsilyl ester	40.43	1348	0.07	55520-95-1
12	Maalialcohol	41.42	1357	0.05	527-90-2
13	α-Bisabolol	41.73	1359	0.36	515-69-5
14	Tetradecanoic acid, trimethylsilyl ester	45.34	1390	3.57	18603-17-3
15	2-Heptadecanone	47.44	1413	0.05	2922-51-2
16	n-Pentanoic acid, trimethylsilyl ester	48.85	1435	0.15	74367-22-9
17	Palmitoleic acid	50.43	1458	55.41	373-49-9
18	Palmitelaidic acid, TMS	53.31	1501	15.85	1206693-35-7
19	6-Pentadecenoic acid,13-methyl-, (6Z)-	57.57	1555	0.08	682751-34-4
20	Linolenic acid	60.68	1594	4.02	463-40-1
21	Ethyl linolenate	61.88	1615	0.41	1191-41-9
22	Oleic acid	63.88	1654	0.12	112-80-1
23	Emulphor	64.77	1671	0.08	5353-25-3
24	2-Nonadecanone	67.83	1742	0.05	629-66-3
25	Glyceryl palmitate	69.64	1790	0.31	542-44-9
26	2-Bromooctadecanal	71.44	1848	0.03	56599-95-2
27	Docosanol	73.04	1901	3.83	661-19-8
28	2-Phenyl-1,3-dithiane	75.22	1985	0.61	5425-44-5
29	1-Tetracosanol	77.16	2059	4.20	506-51-4
30	10-Hydroxy-1,6-dimethyl-9-(propan-2-yl)-5,12-dioxatricyclo [9.1.0.04,6]dodecan-8-yl-3-phenylprop-2-enoate (isomer 2)	78.84	2119	0.30	None
31	(E,E,E,E)-Squalene	79.09	2126	0.25	7683-64-9
32	Dotriacontane	80.42	2168	0.08	544-85-4
33	22-Tricosenoic acid	80.54	2172	1.40	65119-95-1
34	17-[5-Hydroxy-6-(2-hydroxypropan-2-yl)oxan-3-yl]-4,4,10,13,14-pentamethyl-1,2,5,6,9,11,12,15,16,17-decahydrocyclopenta[a]phenanthren-3-one, 2TMS	82.25	2220	0.58	None
35	9,10 DIDEUTERO OCTADECANAL	83.09	2241	0.12	56554-44-0
36	Tocopherols	83.58	2253	0.08	7616-22-0
37	Quercetin 7,3’,4’-trimethyl ether	84.07	2265	0.05	6068-80-0
38	Benzoic acid;tetracosan-1-ol	87.9	2392	0.20	103569-99-9
39	Betulin	90.68	2570	0.20	473-98-3
40	Lupeol	91.67	2698	0.48	545-47-1
Total Identified (%)				100.00	

^1^ RT: Retention time, ^2^ RI: Retention indices determined using a DB-5MS capillary column.

## Data Availability

Data is contained within the article.

## Sample Availability

Samples of the compounds are not available from the authors.
